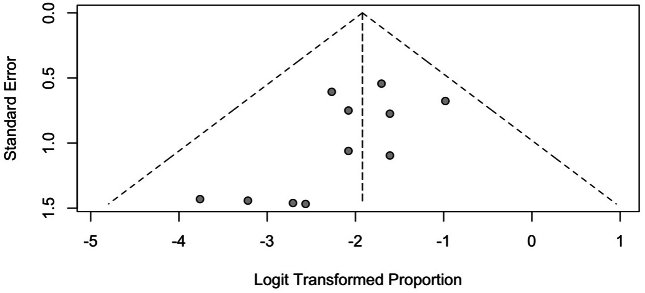# Surgical treatment of post-traumatic elbow stiffness in pediatric patients: a systematic review and meta-analysis

**DOI:** 10.1016/j.xrrt.2025.100646

**Published:** 2025-12-24

**Authors:** Roya Khorram, Areeb Ahmad, Reza Vafadar, Ronald Joseph Shamus, Surena Namdari, G. Russell Huffman, Amir R. Kachooei

**Affiliations:** a Rothman Orthopaedics Florida at AdventHealth, Orlando, FL, USA; b Department of Orthopedic Surgery, Kerman University of Medical Sciences, Kerman, Iran; c The Rothman Institute of Orthopaedics at Thomas Jefferson University Hospital, Philadelphia, PA, USA

**Keywords:** Post-traumatic elbow stiffness, Contractures, Arthroscopic, Open arthrolysis, Adolescents, Teenagers

## Abstract

**Background:**

Post-traumatic elbow stiffness (PTES) has been reported in up to 21% of pediatric patients following elbow fractures and can profoundly compromise a child's function, and independence. The primary surgical approaches include open and arthroscopic arthrolysis, both of which have demonstrated improvements in range of motion (ROM) and functional scores. This systematic review and meta-analysis aims to compare the effectiveness, ROM, and complication rates between open and arthroscopic release in pediatric patients with PTES.

**Methods:**

The Preferred Reporting Items for Systematic Reviews and Meta-Analyses guidelines were utilized to conduct a systematic review and meta-analysis on surgical treatment for PTES. Comprehensive search was conducted in PubMed, Web of Science, Medline, and Scopus from their inception to May 10, 2025. A total of 3,660 records were screened, of which 11 studies were included using open or arthroscopic pediatric PTES elbow arthrolysis. Outcome variables were changes in the ROM and complication rates. Surgical techniques were grouped as arthroscopic vs. open arthrolysis.

**Results:**

Our findings showed that open arthrolysis resulted in greater ROM compared to arthroscopic arthrolysis (open arthrolysis (standardized mean difference: 58°, 95% confidence interval (CI): 46-69; *P* < .001), arthroscopic arthrolysis: (standardized mean difference: 33°, 95% CI: 26-39; *P* = .9)). Regarding the postoperative complications, there was no statistically significant difference between open and arthroscopic arthrolysis (open arthrolysis: [rate: 14%; 95% CI: 9%-22%], arthroscopic arthrolysis: [rate: 7%; 95% CI: 2%-25%].

**Conclusion:**

Open arthrolysis significantly improves ROM in pediatric PTES, outperforming arthroscopic procedures, which showed nonsignificant gains. Complication rates were low and comparable, supporting the safety of both techniques. These findings favor open arthrolysis in severe cases and highlight the need for larger prospective studies to refine arthroscopic indications.

Fractures, dislocations, and prolonged immobilization of the elbow can cause scarring and tissue tightening inside the joint, leading to a loss of motion known as elbow stiffness.^24^ Post-traumatic elbow stiffness (PTES) has been reported in up to 21% of pediatric patients following elbow fractures.^22^ It is typically defined as an active flexion of less than 120° or an extension deficit greater than 30°.^7^ In a cohort of 28 healthy adolescents, the functional arc of elbow motion required to perform contemporary tasks ranged from 40 to 148° of elbow extension/flexion, with tasks like using a phone requiring up to 148° of flexion.^23^ With motion demands this high, PTES can profoundly compromise a child's function, independence, and quality of life when considering the life span and demands.^21^

Nonoperative treatment after elbow trauma is typically favored in children due to their greater healing and remodeling capacity compared to adults.^14^ However, if nonoperative treatment fails to produce greater than 10° of motion gain after 3-6 months, surgical intervention is considered.^21^

The surgical approaches include open and arthroscopic arthrolysis, both of which have demonstrated improvements in range of motion (ROM) and functional scores, with treatment success rates exceeding 88%.^10^^,^^19^^,^^20^ A recent meta-analysis by Onggo et al found that both open and arthroscopic arthrolysis improved ROM and function in children, with fewer complications in the arthroscopic group.^17^ However, the analysis evaluated complications between traumatic and nontraumatic cases, and surgical approach comparisons were limited by the inclusion of only 1 arthroscopic study, as the literature search was restricted to studies published before 2020.

This systematic review and meta-analysis aims to compare the effectiveness, ROM, and complication rates between open and arthroscopic release in pediatric patients with PTES. Our null hypothesis is that there is no significant difference in ROM change and complication rates between open and arthroscopic techniques.

## Methods

The Preferred Reporting Items for Systematic Reviews and Meta-Analyses guidelines was used.

### Search strategy

Two researchers (R.V. and R.S.) systematically searched PubMed, Web of Science, Medline, and Scopus from inception until May 10, 2025. Additionally, 100 pages of Google Scholar were manually queried to include potentially relevant studies. Gray literature was not checked in this study. Medical subject headings and combined truncated key terms were used to construct search strategy queries. The systematic search strategy was designed according to the population, intervention, comparison, outcome framework to assess the outcomes of 3 different surgical procedures frequently employed to treat PTES. These outcomes include the changes in range of motion (ROM), Mayo Elbow Performance Index (MEPI), and visual analog scale. The search strategy used a combination of keywords and controlled vocabulary for the concepts shown in Supplementary Figure S1.

### Screening and eligibility criteria

**Figure 1:**
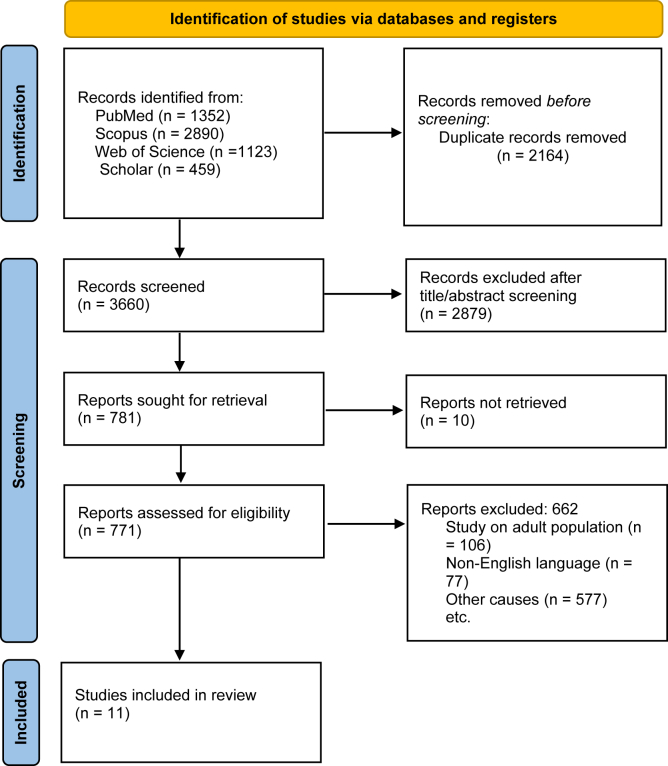
PRISMA flow diagram. *PRISMA*, Preferred Reporting Items for Systematic Reviews and Meta-Analyses.

Two authors (R.V. and R.S.) independently screened titles and abstracts. Duplicates, non-English papers, case reports, and biomechanical/cadaveric studies were excluded. The full texts of the potentially eligible articles were then screened, considering the inclusion and exclusion criteria. The inclusion criteria were as follows: (1) studies on pediatric human subjects with an age below 21, (2) studies evaluating the surgical treatment of PTES, and (3) case series, cohorts, and clinical trials. The exclusion criteria were as follows: (1) non-English studies, (2) studies concerning revision operations, (3) studies on adult patients, (4) nontraumatic stiffness such as burns, cerebral palsy, and brachial plexus injuries, and (5) animal studies, review articles, technical notes, book chapters, conference abstracts, and letters to the editor. Disagreements were resolved by consensus, and discussion with the senior author (A.R.K.). The Preferred Reporting Items for Systematic Reviews and Meta-Analyses flowchart in Figure 1 shows the screening process.

### Methodological quality assessment

Two investigators (A.A. and R.V.) independently assessed the quality of each included study. The Newcastle-Ottawa Scale assessment for risk of bias was applied to assess methodological quality.

### Data extraction

Three reviewers (R.V., A.A., and R.S.) separately collected data from the full texts of the studies using a predesigned Excel sheet. The same reviewers compared and double-checked results, and discrepancies were resolved by consensus and discussion with the senior author (A.R.K.).

### Statistical analysis

The interventions were compared via quantitative meta-analysis using a random effects model to calculate the 95% confidence interval (CI). The null hypothesis that the true effect size is 0 was rejected if the *P* value was less than 0.05. To discern the balance between sampling error and true effect, we conducted a heterogeneity assessment using RStudio, focusing on the computation of I^2^. I^2^ characterizes the percentage of overall variation across studies that results from heterogeneity rather than chance. Negative I^2^ values are adjusted to 0, thereby constraining the I^2^ range between 0% and 100%. An I^2^ value of 0% indicates the absence of observed heterogeneity, while larger values indicate escalating heterogeneity. The heterogeneity of I^2^ > 50% guided our decision to use the random-effect model rather than the fixed-effect model in our meta-analysis, which accounts for the heterogeneity factor. The mean postoperative values were subtracted from the corresponding mean preoperative values to calculate the delta (Δ) ROM, MEPI, and visual analog scale scores. R-Studio was used to perform the meta-analysis.

## Results

### Study selection

A total of 5,824 articles were yielded from the initial search. After exclusion of duplicates, 3,660 articles remained, of which 2,879 were excluded after title/abstract screening. Following the full-text review, 10 retrospective studies, and 1 prospective study, all of which were single-arm investigations evaluating either arthroscopic or open arthrolysis, were included in the quantitative synthesis.^1^^,^^3^, ^4^, ^5^, ^6^, ^7^^,^^12^^,^^13^^,^^15^^,^^16^^,^^18^ (Table I)

**Table I:** Demographic characteristics of eligible studies.

Author, yr	Study type	Age: mean (range)	Patient (n)	Surgery type	Mean follow-up (mo)
Hilgersom,^6^ 2024	Retrospective	17 (14.9-15.9)	6	Arthroscopic	≥2
Kang,^7^ 2023	Retrospective	11 (5.8-14)	21	Open	39
Micheloni,^12^ 2021	Retrospective	15 (8-18)	12	Arthroscopic	67
Aldridge,^1^ 2020	Retrospective	17 (12-21)	18	Open	31
Piper,^17^ 2019	Retrospective	14 (7-20)	26	Open	42
Nowotny,^15^ 2018	Retrospective	14 (11-17)	7	Arthroscopic	45
Ek,^5^ 2016	Retrospective	14 (5-18)	32	Open	66
Darlis,^4^ 2006	Retrospective	17 (13-21)	12	Open	19
Ofiaeli1,^16^ 2001	Prospective	(6-22)	9	Open	6
Bae,^3^ 2001	Retrospective	16 (12.5-20.8)	13	Open	30
Mih,^13^ 1994	Retrospective	13 (5-16)	6	Open	20

### Bias and quality assessment

Ten observational cohort studies were evaluated using the Newcastle-Ottawa Scale. Of these, 8 studies (80%) were rated as high quality (≥7 points), and 2 studies (20%) were rated as moderate quality (4–6 points) (Supplementary Table S1). Common limitations among moderate-quality studies included a lack of a control group and limited adjustment for confounding variables.

The single case series included in this review was assessed using the National Institutes of Health Quality Assessment Tool for Case Series Studies and was rated as good quality, with clearly defined objectives, consistent outcome measures, and adequate follow-up (Supplementary Table S2).

### Study characteristics

All 11 included studies were published between 1994 and 2024, comprising a total of 162 pediatric patients who underwent surgery for PTES. The mean patient age across studies ranged from 11 to 17 years (Table I). Three studies evaluated arthroscopic arthrolysis, while 8 focused on open arthrolysis as treatment modalities. Among the 11 studies, anterior capsular release was performed in 8, whereas 3 described only posterior release.^6^^,^^12^^,^^16^ Posterior release and adjunctive bony procedures, including osteophyte débridement, heterotopic ossification excision, and olecranon or coronoid fossa work, were described in all studies. Reported follow-up durations after surgery ranged from 2 to 67 months. All included studies reported preoperative and postoperative ROM, as well as complication outcomes. Delta ROM values and complications for each study are presented in Tables II and III.

**Table II:** Included studies for ROM based on surgical method.

Author, yr	Surgery type	Mean ROM (delta)	SD (delta ROM)
Hilgersom,^6^ 2024	Arthroscopic	27	32.91
Kang,^7^ 2023	Open	78.3	15.07
Micheloni,^12^ 2021	Arthroscopic	33.9	23.63
Aldridge,^1^ 2020	Open	37	25.37
Piper,^17^ 2019	Open	48	31.37
Nowotny,^15^ 2018	Arthroscopic	33	19.94
Ek,^5^ 2016	Open	54	24.62
Darlis,^4^ 2006	Open	54	23.65
Ofiaeli1,^16^ 2001	Open	71.8	7.528
Bae,^3^ 2001	Open	54	30.34
Mih,^13^ 1994	Open	60.84	27.88

*ROM*, range of motion; *SD*, standard deviation.

**Table III:** Included studies for complications based on surgical method.

Author, yr	Patients	Surgery type	Total complications (n)	Complication type
Hilgersom,^6^ 2024	6	Arthroscopic	0	None
Kang,^7^ 2023	21	Open	0	None
Micheloni,^12^ 2021	12	Arthroscopic	1	Heterotopic ossification (n = 1)
Aldridge,^1^ 2020	18	Open	2	Posterior interosseous nerve palsy (n = 1, resolved), lateral antebrachial cutaneous nerve palsy (n = 1, persistent, nonlimiting)
Piper,^17^ 2019	26	Open	4	Recurrent contracture (n = 2), ulnar neuropathy (n = 2)
Nowotny,^15^ 2018	7	Arthroscopic	0	None
Ek,^5^ 2016	32	Open	3	Deep infection (n = 1, resolved), subcutaneous hematoma (n = 1), iatrogenic humeral shaft fracture (n = 1, nonoperative)
Darlis,^4^ 2006	12	Open	3	Superficial wound infection (n = 1) heterotopic ossification (n = 1)
Ofiaeli1,^16^ 2001	9	Open	0	None
Bae,^3^ 2001	11	Open	3	Wound drainage requiring I&D (n = 1), transient ulnar neuropraxia (n = 1), pain (n = 1)
Mih,^13^ 1994	6	Open	0	None

*I&D*, incision and drainage.

### Range of motion

We included 11 studies in our analysis: 3 (n = 25) evaluated arthroscopic arthrolysis and 8 (n = 137) evaluated open arthrolysis in pediatric patients with PTES. ROM was assessed with a goniometer in 9 studies, whereas 2 did not specify the measurement technique.^3^^,^^6^ Due to considerable heterogeneity (I^2^ = 91%), a random-effects model was applied. Overall, surgical treatment significantly improved ROM [standardized mean difference (SMD): 51°, 95% CI: 40-62; *P* < .0001].

**Figure 2:**
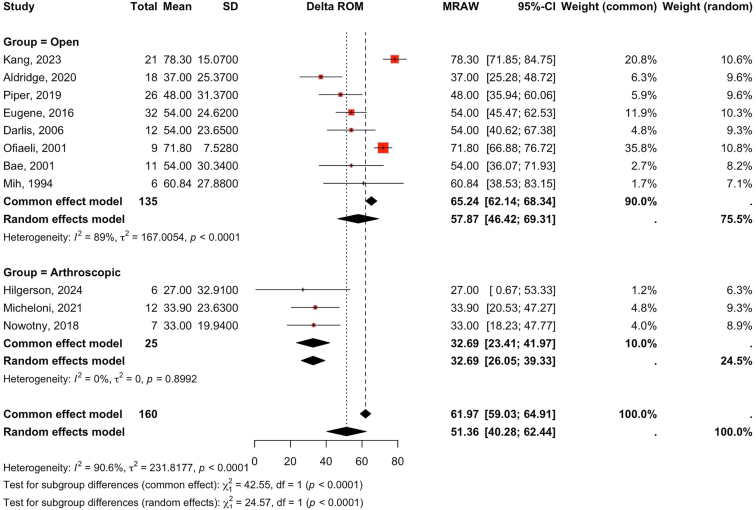
Forest plot comparing change in ROM after open vs. arthroscopic arthrolysis. *ROM*, range of motion.

Subgroup analysis demonstrated that open arthrolysis resulted in a significant improvement in ROM (SMD: 58°, 95% CI: 46-69; *P* < .0001), whereas arthroscopic arthrolysis did not yield a statistically significant change (SMD: 33°, 95% CI: 26-39; *P* = .9). Direct comparison confirmed that open arthrolysis resulted in greater ROM gains than arthroscopic arthrolysis (SMD: 58° vs. 33°; *P* < .01) (Fig. 2).

### Complications

**Figure 3:**
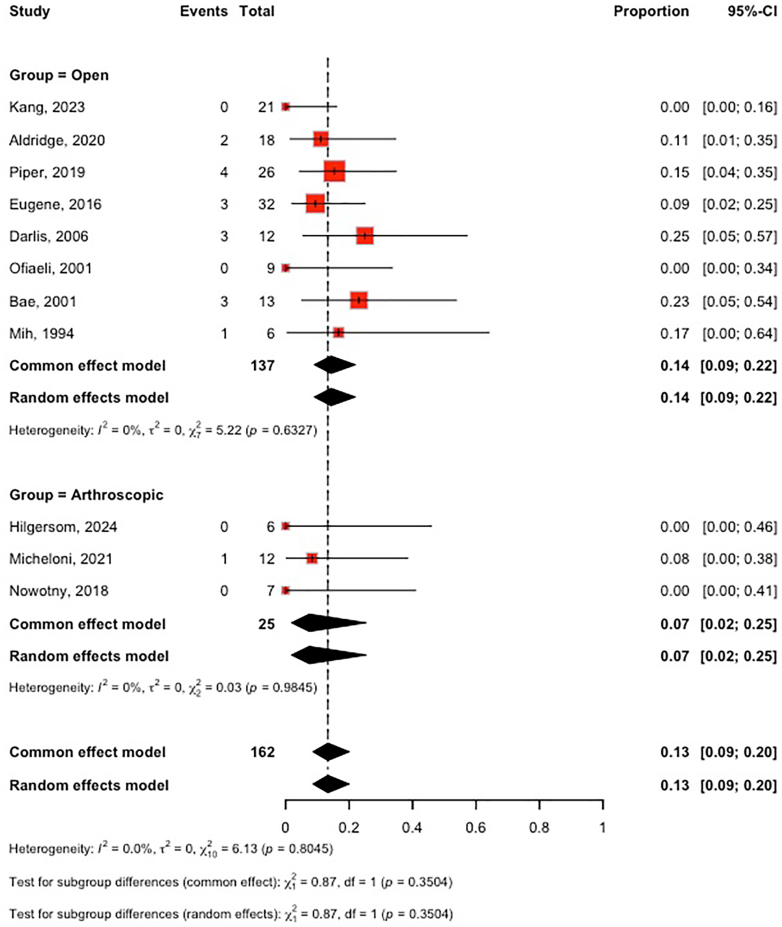
Forest plot comparing complication rates after open vs. arthroscopic arthrolysis.

Reporting of complications varied considerably between studies, with outcomes such as stiffness, nerve injury, heterotopic ossification, and infection inconsistently defined. Because of this heterogeneity and the limited number of events per category, statistical subgroup analysis was not feasible, and all complications were therefore pooled into a single outcome for analysis. We included all 11 studies in the analysis of total complications: 3 studies (n = 25) evaluated arthroscopic arthrolysis and 8 studies (n = 137) evaluated open arthrolysis in pediatric patients with PTES. Due to the low heterogeneity between studies (I^2^ = 0%), a fixed-effect model was applied. Our analysis revealed no significant difference between open and arthroscopic arthrolysis (open arthrolysis: [rate: 14%; 95% CI 9%-22%] vs. arthroscopic arthrolysis: [rate: 7%; 95% CI 2%-25%]. Other complications were also reported across the included studies. In the open arthrolysis group, recurrent contracture occurred in 2 patients,^18^ subcutaneous hematoma in 1 patient, iatrogenic humeral shaft fracture (managed nonoperatively) in 1 patient,^5^ pain and wound drainage requiring incision and drainage in 1 patient.^3^ Heterotopic ossification was also noted in 1 case within the open group.^4^ In the arthroscopic group, heterotopic ossification was reported in 1 case.^12^ (Fig. 3)

### Publication bias

**Supplementary Figure S2:**
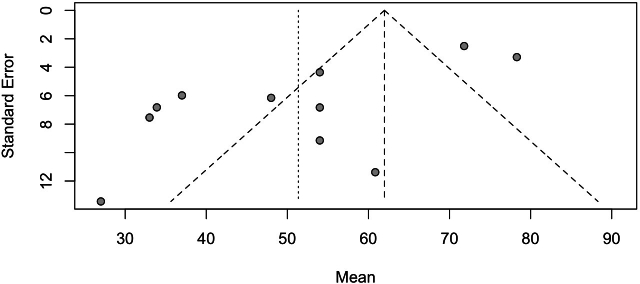


Funnel plots were visually inspected to assess publication bias for the 2 primary outcomes: ROM (11 studies) and complications (11 studies). Both plots appeared symmetrical, with no substantial visual asymmetry, suggesting a low risk of publication bias (Supplementary Figs. S2 and S3).

## Discussion

The findings of this study indicate that open arthrolysis is associated with superior gains in ROM compared to arthroscopic procedures (58° vs. 33°). In contrast, complication rates, although higher in the open technique (14% vs. 7%), showed no significant differences.

These findings are consistent with the literature, where open approaches have been associated with more substantial improvements, particularly in cases of severe or chronic stiffness.^7^ Kang et al^7^ reported excellent outcomes using open arthrolysis in 21 children with chronic elbow dislocation and severe stiffness, achieving a mean postoperative ROM of 119° ± 15°, compared to a preoperative mean of 18° ± 8.3°, and an 81% excellent outcome rate based on MEPI. Their results underscore the effectiveness of the open approach in more complex cases, especially where extensive intra-articular pathology or bony impingement exists.

Multiple factors can influence the outcomes of open surgical release, and this remains a topic of discussion. Ek et al^5^ analyzed 32 pediatric patients who underwent open arthrolysis, of whom 30 were PTES. Their results showed a mean of 54° gain in ROM with a significant change that was maintained over a mean follow-up of 5.5 years. They found no significant correlation between the final ROM and the complexity of the fractures. Moreover, they recommend a lower threshold for ulnar nerve transposition in severe flexion contractures, which they did in 66% of their patients.

Our study found no significant improvement in ROM following arthroscopic release in pediatric patients with PTES. Similar to our findings, Nowotny et al^15^ evaluated elbow arthroscopy in a pediatric cohort with diverse indications, including PTES. While they reported good-to-excellent outcomes in 82% of patients overall, subgroup analysis revealed that the gain in ROM in this subgroup was not statistically significant, increasing from 123° ± 18° to 132° ± 15° (*P* = .66). Likewise, the improvement in MEPI scores was not statistically significant in the post-traumatic group. In contrast, Micheloni et al^12^ reported a significant improvement in ROM in 12 pediatric patients with PTES, including a mean increase of 22° in flexion (*P* < .001) and 20.° in extension (*P* < .001) at a mean follow-up time of 67 months. Similarly, Hilgersom et al^6^ reported a significant improvement (median: 23°; interquartile range: 15°-30°) in ROM in a subgroup of 6 patients who underwent PTES release. These discrepancies may stem from several factors. Differences in sample size may influence statistical power, with larger PTES-specific cohorts such as Micheloni's study providing more robust estimates of treatment effect. Additionally, variation in follow-up duration, patient selection, and severity of stiffness may impact measured outcomes, as more severe contractures or complex post-traumatic pathology are less likely to show equivalent gains.^12^ Finally, surgical and rehabilitation differences, including the extent of capsular release and adherence to postoperative therapy, could also account for variation across studies. Despite these variations, complication rates remained low across all arthroscopic studies, supporting the safety of arthroscopic release in pediatric PTES.^6^^,^^12^^,^^15^

The differences in outcomes between open and arthroscopic arthrolysis may be explained by several factors. Open arthrolysis allows more extensive capsulotomy and capsulectomy, removal of both intracapsular and extracapsular heterotopic ossification, and direct visualization of extra-articular pathologies. Additionally, selection bias may contribute to the differences observed between surgical techniques. Arthroscopic release is often reserved for less severe contractures with relatively preserved preoperative motion, which can limit the degree of measurable improvement. In contrast, open arthrolysis is typically performed in cases with more substantial stiffness. Therefore, the greater delta ROM observed in the open cohort may reflect differences in baseline severity rather than surgical technique alone.^11^ Kang et al^7^ highlighted that open arthrolysis using a combined medial and lateral approach allowed for thorough removal of fibrotic tissue and osteophytes, which is often necessary in pediatric patients with long-standing or severe stiffness.

Our meta-analyses revealed differing levels of heterogeneity across outcomes. For complication rates, statistical heterogeneity was negligible (I^2^ = 0%), indicating strong consistency among the included studies. In contrast, the ROM analysis revealed substantial heterogeneity (*I*^*2*^ = 91%). Several factors may have contributed to this variability, including differences in study design, surgical approach (arthroscopic vs. open), postoperative rehabilitation protocols, and patient characteristics. Variations in the indication for surgery (PTES following operative vs. nonoperative treatment) and preoperative severity of elbow stiffness likely also influenced the observed outcomes.

When comparing the results of the present study to the findings of a previous study on the adult population, the gain in ROM following open arthrolysis was quite similar between pediatric and adult populations. The pediatric population showed an average ROM gain of 58°, closely matching the 60° improvement reported in adults. This similarity suggests that open arthrolysis is consistently effective across age groups.^8^

In contrast, the results for arthroscopic arthrolysis revealed a notable difference. In the previous study on the adult population, a greater ROM improvement of 47° was observed, compared to 33° improvement in pediatric patients in the present study.^8^ The complexity and unique challenges associated with performing elbow arthroscopy in pediatric patients are emphasized by Kircher. It is highlighted that the pediatric elbow joint is anatomically smaller and more complex, necessitating specialized training and advanced technical skills. Additionally, the proximity of neurovascular structures is noted to render the procedure more technically demanding and potentially riskier compared to arthroscopy in larger joints such as the knee or shoulder.^9^

As highlighted in the Andelman et al study, PTES often involves more complex pathologies, especially in patients who have undergone previous surgical interventions. This complexity can lead to a higher complication rate compared to other indications for elbow arthroscopy in children.^2^ Finally, it is important to note that the sample size for pediatric arthroscopic release is smaller compared to adult studies. This difference in sample size can impact the statistical power of the results and should be taken into consideration when interpreting the findings.

Regarding complications, although the mean rate in the arthroscopic technique (7%) was almost half of the open release (14%), we could not show a statistically significant difference, which is consistent with the findings of the adult population study. This suggests that while the type of procedure may not significantly impact the overall complication rate, the inherent complexities of pediatric cases, especially in post-traumatic scenarios, remain a crucial consideration.^8^

This systematic review and meta-analysis has several limitations. First, the overall number of studies specifically addressing pediatric patients with PTES, particularly those treated arthroscopically, was limited, resulting in small subgroup sample sizes and reduced statistical power for comparative analyses. Additionally, substantial heterogeneity was observed in the ROM outcomes (I^2^ = 91%), likely reflecting differences in study design, surgical techniques, follow-up durations, rehabilitation protocols, and patient selection, all of which may have influenced the pooled estimates. Furthermore, although quality assessment tools were used, variability in methodological rigor across studies remains a concern, particularly regarding the standardization of outcome reporting and definitions of complications. Lastly, as a level IV study compiling primarily retrospective case series, there is a high risk of inherent biases, such as selection bias and publication. These factors make it challenging to generate definitive conclusions and highlight the need for prospective, high-quality, and PTES-specific trials in the pediatric population.

## Conclusion

Our study demonstrates that open arthrolysis significantly enhances ROM in pediatric PTES, achieving superior gains compared to arthroscopic procedures, which showed some ROM improvement but did not reach statistical significance. Complication rates remain comparable and low across techniques, affirming their safety in children despite anatomical complexities. These results support prioritizing open arthrolysis for severe or chronic cases, while advocating for future prospective studies with larger cohorts to better delineate indications and refine arthroscopic protocols for optimal long-term function.

## Disclaimer

Funding: No funding was disclosed by the authors.

Conflicts of interest: The authors, their immediate families, and any research foundation with which they are affiliated have not received any financial payments or other benefits from any commercial entity related to the subject of this article.

## Figures and Tables

**Supplementary Figure S3:**