# Supplementary material for: Surgical treatment of post-traumatic elbow stiffness in pediatric patients: a systematic review and meta-analysis
Source: JSES Rev Rep Tech. 2025 Dec 24;6(2):100646. doi: 10.1016/j.xrrt.2025.100646 (PMC12876576; doi:10.1016/j.xrrt.2025.100646)
Supplement: Supplementary Figure S1 [file mmc1.docx]

Supplementary figure 1. PubMed Search result

| **PubMed Search on 2024-01-20** | | **Results** |
| --- | --- | --- |
| **#5** | ((((trauma*[Title/Abstract]) OR (wound*[Title/Abstract])) OR (injur*[Title/Abstract])) AND (elbow[Title/Abstract])) AND (((((stiff*[Title/Abstract]) OR (contracture[Title/Abstract])) OR (ankylos*[Title/Abstract])) OR (rigid*[Title/Abstract])) OR ("limitation of motion"[Title/Abstract])) | [1,204](https://pubmed.ncbi.nlm.nih.gov/?term=%28%28%28%28trauma%2A%5BTitle%2FAbstract%5D%29+OR+%28wound%2A%5BTitle%2FAbstract%5D%29%29+OR+%28injur%2A%5BTitle%2FAbstract%5D%29%29+AND+%28elbow%5BTitle%2FAbstract%5D%29%29+AND+%28%28%28%28%28stiff%2A%5BTitle%2FAbstract%5D%29+OR+%28contracture%5BTitle%2FAbstract%5D%29%29+OR+%28ankylos%2A%5BTitle%2FAbstract%5D%29%29+OR+%28rigid%2A%5BTitle%2FAbstract%5D%29%29+OR+%28%22limitation+of+motion%22%5BTitle%2FAbstract%5D%29%29&ac=no&sort=relevance) |
| **#4** | ((((stiff*[Title/Abstract]) OR (contracture[Title/Abstract])) OR (ankylos*[Title/Abstract])) OR (rigid*[Title/Abstract])) OR ("limitation of motion"[Title/Abstract]) | [245,207](https://pubmed.ncbi.nlm.nih.gov/?term=%28%28%28%28stiff%2A%5BTitle%2FAbstract%5D%29+OR+%28contracture%5BTitle%2FAbstract%5D%29%29+OR+%28ankylos%2A%5BTitle%2FAbstract%5D%29%29+OR+%28rigid%2A%5BTitle%2FAbstract%5D%29%29+OR+%28%22limitation+of+motion%22%5BTitle%2FAbstract%5D%29&ac=no&sort=relevance) |
| **#3** | (((stiff*[Title/Abstract]) OR (contracture[Title/Abstract])) OR (ankylos*[Title/Abstract])) OR (rigid*[Title/Abstract]) | [244,848](https://pubmed.ncbi.nlm.nih.gov/?term=%28%28%28stiff%2A%5BTitle%2FAbstract%5D%29+OR+%28contracture%5BTitle%2FAbstract%5D%29%29+OR+%28ankylos%2A%5BTitle%2FAbstract%5D%29%29+OR+%28rigid%2A%5BTitle%2FAbstract%5D%29&ac=no&sort=relevance) |
| **#2** | ((trauma*[Title/Abstract]) OR (wound*[Title/Abstract])) OR (injur*[Title/Abstract]) | [1,521,784](https://pubmed.ncbi.nlm.nih.gov/?term=%28%28trauma%2A%5BTitle%2FAbstract%5D%29+OR+%28wound%2A%5BTitle%2FAbstract%5D%29%29+OR+%28injur%2A%5BTitle%2FAbstract%5D%29&ac=no&sort=relevance) |
| **#1** | elbow[Title/Abstract] | [39,309](https://pubmed.ncbi.nlm.nih.gov/?term=elbow%5BTitle%2FAbstract%5D&ac=no&sort=relevance) |

| **Web of Science Search on 2024-01-20** | | **Results** |
| --- | --- | --- |
| **#4** | #1 AND #2 AND #3 | [1,123](https://www.webofscience.com/wos/woscc/summary/28361406-4c8e-42ed-b8bd-d405b9ed9c76-c6fc7414/relevance/1) |
| **#3** | ((((TS=(stiff*)) OR TS=(contracture)) OR TS=(ankylos*)) OR TS=(rigid*)) OR TS=("Limitation of motion") | [560,438](https://www.webofscience.com/wos/woscc/summary/b9ccc932-8d8c-4856-b759-854837b6d852-c6fc737c/relevance/1) |
| **#2** | ((TS=(trauma*)) OR TS=(wound)) OR TS=(injur*) | [1,933,058](https://www.webofscience.com/wos/woscc/summary/97df8813-2996-42f6-9a47-cd69f219c3a5-c6fc6eb4/relevance/1) |
| **#1** | TS=(elbow) | [41,390](https://www.webofscience.com/wos/woscc/summary/9ec0810e-0713-4d93-9919-5afc729c3de6-c6fc725c/relevance/1) |

| **Scopus Search on 2024-01-20** | | **Results** |
| --- | --- | --- |
| **#4** | ( TITLE-ABS-KEY ( trauma* ) OR TITLE-ABS-KEY ( wound ) OR TITLE-ABS-KEY ( injur* ) ) AND ( TITLE-ABS-KEY ( elbow ) ) AND ( TITLE-ABS-KEY ( stiff* ) OR TITLE-ABS-KEY ( contracture ) OR TITLE-ABS-KEY ( ankylos* ) OR TITLE-ABS-KEY ( rigid* ) OR TITLE-ABS-KEY ( "limitation of motion" ) ) | [2,650](https://www-scopus-com.ezproxy.udes.edu.co/search/history/results.uri?origin=searchhistory&shid=5) |
| **#3** | TITLE-ABS-KEY ( stiff* ) OR TITLE-ABS-KEY ( contracture ) OR TITLE-ABS-KEY ( ankylos* ) OR TITLE-ABS-KEY ( rigid* ) OR TITLE-ABS-KEY ( "limitation of motion" ) | 963,803 |
| **#2** | TITLE-ABS-KEY ( trauma* ) OR TITLE-ABS-KEY ( wound ) OR TITLE-ABS-KEY ( injur* ) | 2,742,350 |
| **#1** | TITLE-ABS-KEY ( elbow ) | 75,683 |
