# Supplementary material for: Surgical treatment of post-traumatic elbow stiffness in pediatric patients: a systematic review and meta-analysis
Source: JSES Rev Rep Tech. 2025 Dec 24;6(2):100646. doi: 10.1016/j.xrrt.2025.100646 (PMC12876576; doi:10.1016/j.xrrt.2025.100646)
Supplement: Supplementary Table S1 [file mmc2.docx]

**Supplementary Table 1. Quality Assessment of the Retrospective Cohort Studies Using the Newcastle-Ottawa Scale**

| Author | Selection (Max 4★) | Comparability (Max 2★) | Exposure/Outcome (Max 3★) | Total Score (Max 9) | Quality Rating |
| --- | --- | --- | --- | --- | --- |
| Hilgersom ^6^, 2024 | ★★★ | ★ | ★★★ | 7 | High |
| Kang^7^, 2023 | ★★★ | ★ | ★★★ | 7 | High |
| Micheloni^12^, 2021 | ★★★ | ★ | ★★★ | 7 | High |
| Aldridge^1^, 2020 | ★★★ | ★ | ★★★ | 7 | High |
| Piper^17^, 2019 | ★★★ | ★ | ★★★ | 7 | High |
| Nowotny^15^, 2018 | ★★★ | ★ | ★★★ | 7 | High |
| Darlis^4^, 2006 | ★★★ | ★ | ★★★ | 7 | High |
| Ofiaeli1^16^, 2001 | ★★★ |  | ★★★ | 6 | Moderate |
| Bae^3^, 2001 | ★★★ | ★ | ★★★ | 7 | High |
| Mih^13^, 1994 | ★★★ |  | ★★★ | 6 | Moderate |
