# Supplementary material for: Surgical treatment of post-traumatic elbow stiffness in pediatric patients: a systematic review and meta-analysis
Source: JSES Rev Rep Tech. 2025 Dec 24;6(2):100646. doi: 10.1016/j.xrrt.2025.100646 (PMC12876576; doi:10.1016/j.xrrt.2025.100646)
Supplement: Supplementary Table S2 [file mmc3.docx]

**Supplementary Table 2. NIH Quality Assessment for Case Series**

| **Author** | **Criteria** | **Response** | **Notes** |
| --- | --- | --- | --- |
| Ek^5^, 2016 | 1. Was the study question or objective clearly stated? | Yes | Clear objective to evaluate long-term outcomes of elbow contracture release in patients <18 years. |
|  | 2. Was the study population clearly and fully described, including a case definition? | Yes | Detailed inclusion criteria, demographics, and diagnoses were provided. |
|  | 3. Were the cases consecutive? | Yes | All eligible patients from 1994–2012 by the senior author were included. |
|  | 4. Were the subjects comparable? | Yes | Although etiologies varied, demographic and baseline data were provided and subgroups analyzed. |
|  | 5. Was the intervention clearly described? | Yes | Surgical technique, postoperative care, and follow-up protocols thoroughly explained. |
|  | 6. Were the outcome measures clearly defined, valid, reliable, and implemented consistently across all study participants? | Yes | Range of motion measured with goniometer; standardized protocol used. |
|  | 7. Was the length of follow-up adequate? | Yes | Mean follow-up was 66 months (range: 7–202), sufficient to assess durability of outcomes. |
|  | 8. Were the statistical methods well-described? | Yes | T-tests, Analysis of variance (ANOVA), and P values reported; software specified. |
|  | 9. Were the results well-described? | Yes | Comprehensive results with tables, figures, and subgroup analyses. |

**Quality Rating: Good**

**Rater #1 initials:** RK
**Rater #2 initials:** AA
